# Three‐dimensional printed polyether‐ether‐ketone implant for extensive chest wall reconstruction: A case report

**DOI:** 10.1111/1759-7714.13560

**Published:** 2020-07-17

**Authors:** Lei Wang, Xi Liu, Tao Jiang, Lijun Huang

**Affiliations:** ^1^ Department of Thoracic Surgery, Tangdu Hospital The Fourth Military Medical University Xi'an China

**Keywords:** Chest wall reconstruction, polyether‐ether‐ketone, three‐dimensional printed (3DP) implant

## Abstract

Three‐dimensional printed (3DP) implant offers a valid option with perfect anatomic fitting in individual and skeletal reconstruction of the chest wall. Herein, we present the case of a patient with a large chest wall tumor, where an extensive chest wall defect was repaired using 3DP polyether‐ether‐ketone (PEEK) implants. Surgical treatment planning was performed according to the computed tomography (CT) images in DICOM format. A 3DP implant was then design and fabricated. A wide excision of the chest wall tumor was performed, including the entire sternum, 2–6 costal cartilage and ribs, and parietal pleura. Furthermore, a skeletal reconstruction was carried out using a 3DP PEEK implant. The patient recovered well without surgical complications or tumor recurrence in the following year. In general, 3DP PEEK implant is an appropriate alternative for chest wall reconstruction.

**Key points:**

**Significant findings of the study:**

Skeletal reconstruction after wide excision of the chest wall remains a challenging problem for clinicians.

**What this study adds:**

3DP PEEK implant is an appropriate alternative for chest wall reconstruction.

## Graphical abstract

3DP PEEK implant was anchored to the clavicle and remaining ribs using steel wires.

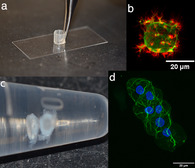

## Introduction

Chest wall reconstruction after wide excision of cancer and surrounding tissues remains a challenging problem in clinical practice due to poor functional outcomes and high respiratory complication rates. Traditional rigid implants such as methylmethacrylate, titanium plates and biological ribs customized by hand during surgery have been previously reported to assist in the relief of respiratory complications.^1^ However, they do not offer a valid option with perfect anatomic fitting. Three‐dimensional printed (3DP) custom‐made implants have been used in recent studies^2^, ^3^ because of their advantages in individual and skeletal reconstruction of the chest wall. Herein, we present a case of a patient with a large chest wall tumor, where the extensive chest wall defect was repaired using 3DP polyether‐ether‐ketone (PEEK) implants.

## Case report

**Figure 1:**
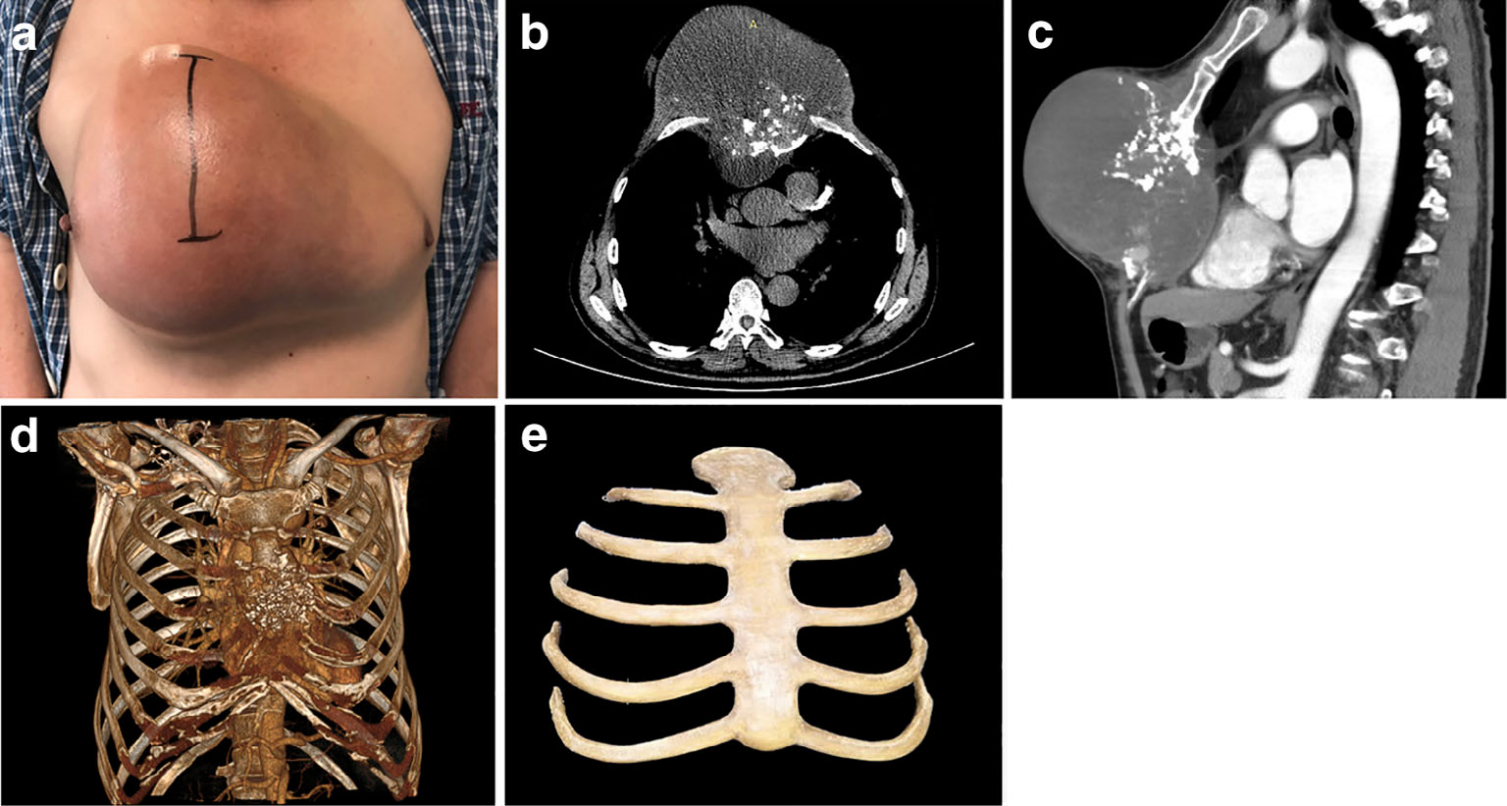
(**a**) A 59‐year‐old male patient presented with a large chest tumor located throughout the anterior chest wall. (**b**) Chest computed tomography (CT) showed the destruction of the sternum and large soft tissue masses. The right pectoralis major muscle was invaded by the tumor. (**c**) Sagittal chest CT showed a large mass had compressed the pericardium. (**d**) Mimics software was used to perform a concrete model of tumor destruction. (**e**) The 3DP PEEK implant weighed 206 g with bending strength at 141 ± 7 MPa and tensile strength at 89 ± 3 MPa, elastic modulus at 2.8 ± 1.5 GPa.

In June 2018, a 59‐year‐old male with a large chest wall tumor was admitted to our department. He was found to have a tumor located in the anterior chest wall which was approximately 25 × 20 × 15 cm in volume (Fig 1a). The pathological results following needle biopsy indicated that it was a chondrosarcoma which is a tumor not receptive to radiotherapy and chemotherapy. Chest computed tomography (CT) images of 1.0 mm slice thickness and a positron emission tomography (PET)‐CT examination were performed to identify distant metastasis in surrounding organs. Significant destruction was identified in the sternum and heart (Fig 1b,c). CT images in DICOM format were exported to Mimics software (version 17.0, Materialize Inc., USA) for surgical planning and showed the destruction which had been caused by the tumor (Fig 1d). The resection margin was predicted to be at least 3 cm from the tumor margin. A 3DP implant was designed to simulate the shape of the patient's chest wall. Surgical grade PEEK materials were purchased from British Victrex Corporation. A fused deposition modeling 3DP machine (Jugao‐AM‐Doctor, Xi'an Jiaotong University, melting temperature 340°C) was used to fabricate the PEEK implant (Fig 1e). In order to protect soft tissue, the prosthesis was polished. It took five days from surgical design for the implant to be ready.

**Figure 2:**
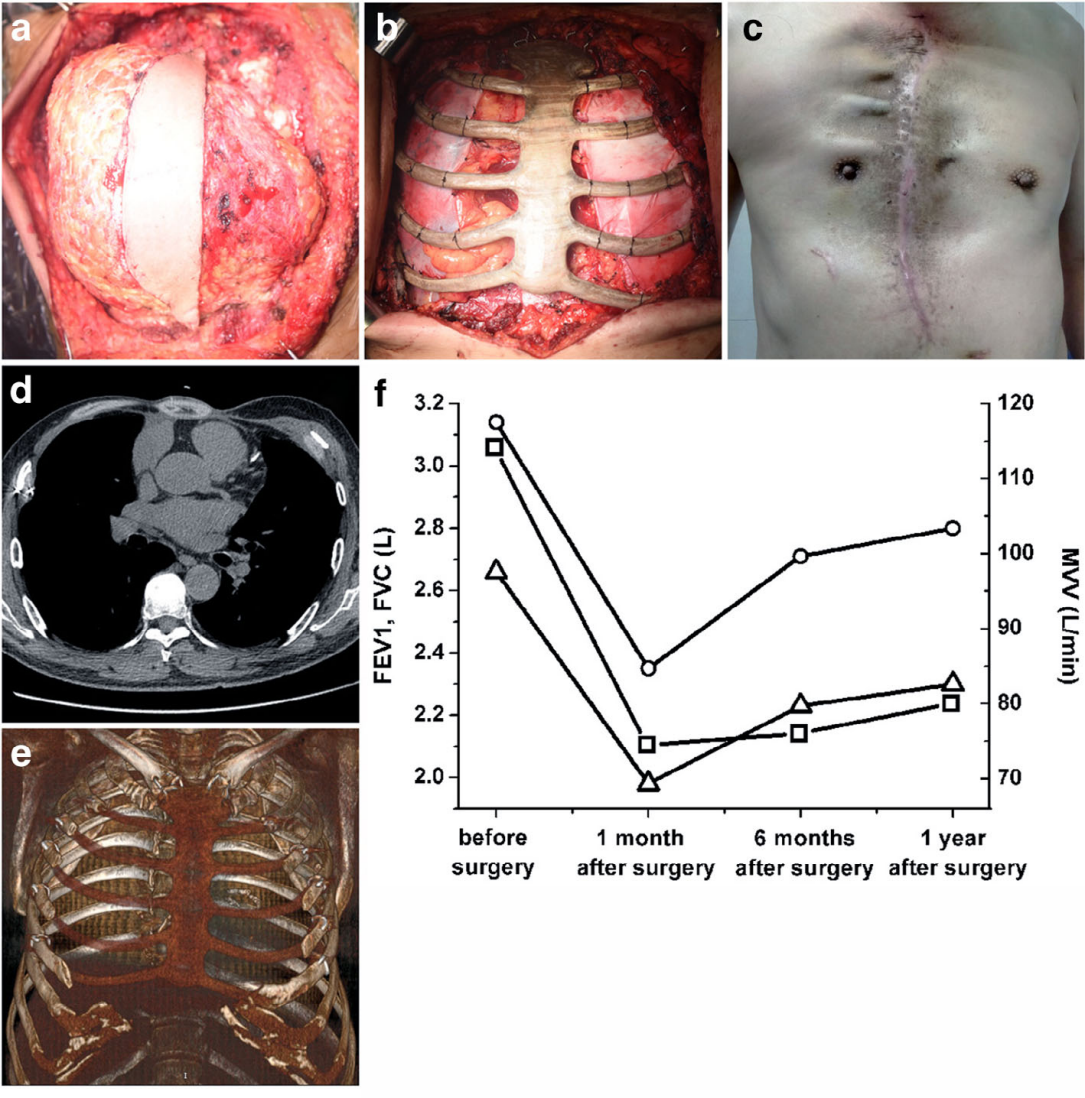
(**a**) The tumor was seen to be invading the sternum, ribs, proximal pleura and muscle. The whole tumor was exposed and part of the skin was left on the surface of the tumor, which needed to be removed with the tumor because it was less than 2 cm away from it. (**b**) A complete en bloc resection of the anterior chest wall was performed and the 3DP polyether‐ether‐ketone (PEEK) implant was anchored to the clavicle and remaining ribs using steel wires. A pericardial patch was densely suspended on the inner surface of the PEEK implant. (**c**) The incision site of the patient one year after surgery. (**d**) Chest computed tomography (CT) scan of the patient one year after surgery. (**e**) Three‐dimensional reconstruction of chest CT images of the patient one year after surgery. (**f**) The changes in pulmonary function values pre‐ and postoperatively, including 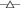, FEV1; 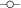, FVC and 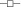, MVV.

A wide excision of the chest wall tumor was performed according to surgical planning (Supplemental Video 1). The entire sternum, 2–6 costal cartilage and ribs, and parietal pleura were removed intraoperatively (Fig 2a). The diaphragm had not been invaded. Based on frozen tissue section analysis at the margin of resection (skin, muscle and subcutaneous adipose tissue), a disease‐free resection margin (R0) was established. The margin of resection was also again confirmed by a definitive histopathological examination (hematoxylin and eosin staining). After wide excision of the tumor, the patient was left with a large defect of 35 × 30 cm minimum in the anterior chest wall. To ensure the stability of the junction parts, the PEEK implant and residual ribs, including the sternoclavicular joint and rib ends, were fixed with steel wires (Fig 2b). A pericardial patch (Guanhao Biotech Corporate, Guangzhou, China) was used to cover the lung and heart after the implant. The edge of the patch was sutured to the remaining pleura. The tumor weighed approximately 3 kg. As the tumor grows, the skin covering the tumor proliferates Moreover, the skin which had been removed seemed to be less due to proliferation. In this situation, residual skin can be used to cover the prothesis without high tension after resection, and the remaining skin after resection was used to cover the external surface of the PEEK implant. Two negative pressure drainage tubes of thoracic wall were retained under the flap during surgery and removed two weeks later **(** Fig S1a,b **)**. The pressure bandage was interleaved at the incision for two weeks. Changes of body temperature were monitored and aseptic procedures were strictly followed when the dressing was changed. The skin flap color, temperature and local blood circulation were observed through the skin flap window. The wound dressing was changed when it oozed, and the wound was kept clean and dry. Maintain the negative pressure drainage of chest wall, and the staffs were asked to observe the pressure in the negative pressure drainage bottle regularly. The increase of negative pressure may cause the backflow of drainage fluid and cause infection. Ceftriaxone was injected intravenously at 0.5 hours and 72 hours postoperatively. Postoperative pathology confirmed the tumor was a chondrosarcoma. The patient recovered well without surgical complications or tumor recurrence in the following year (Fig 2c–e). Pulmonary function tests were performed routinely pre‐ and post‐surgery (Fig 2f), and the FEV1, FVC and MVV values fell to their lowest levels in the first month after surgery. However, all the values increased gradually in the first year after surgery.

## Discussion

There are difficulties which must be taken into consideration during surgical planning of this procedure. First, in the case reported here, the chest wall defect after wide excision was extensive and covered almost the entire anterior chest wall. The rigid implant should maintain chest wall stability without comprising adequate lung function which requires the material to be rigid but not restricting chest wall movement. Otherwise, the lack of respiratory muscles, including ectopectoralis and intercostal muscles, which were completely removed during surgery, may lead to decreased pulmonary function even though bony reconstruction was performed. Titanium prostheses are the most common implants for chest wall reconstruction, but the elasticity modulus of titanium (110 GPa) is much higher than that (12 GPa) of cortical bone.^4^ PEEK material is a promising alternative for titanium in orthopedics. It has been used in clinical practice for 20 years because of its good biocompatibility, biomechanical properties and stability. It also has a close mechanical strength (flexural strength, 141 ± 7 MPa and tensile strength, 89 ± 3 MPa) to the cortical bone and this could minimize the stress‐shielding effect.

In this report, the biggest 3DP PEEK implant in our institute was used to repair the extensive chest wall defect. Although the primary sternum and ribs had been invaded and destroyed by the tumor, the shape of the implant simulated the anatomical structure of the chest wall. In the follow‐up period, this implant was able to provide enough support to maintain the shape of thorax. Previous studies^4^, ^5^ have suggested that 3DP PEEK implant is a safe and effective alternative, especially in preserving the pulmonary function of the patient effectively after surgery. In the first year, the patient recovered enough pulmonary function to live on his own. Additionally, in order to prevent dyspnea after surgery, the patient had been asked to perform abdominal breathing exercises one month before surgery.

In conclusion, 3DP PEEK implant is an appropriate alternative for chest wall reconstruction.

## Disclosure

No authors report any conflict of interest.

## Supporting information


**Supplementary Figure 1** (a) The patient's condition on the first day after surgery; and (b) the condition of the patient on the seventh day after the operation.

**Supplementary Video 1**
