# Supplementary figures and images for: Three‐dimensional printed polyether‐ether‐ketone implant for extensive chest wall reconstruction: A case report
Source: Thorac Cancer. 2020 Jul 17;11(9):2709–12. doi: 10.1111/1759-7714.13560 (PMC7471033; doi:10.1111/1759-7714.13560)

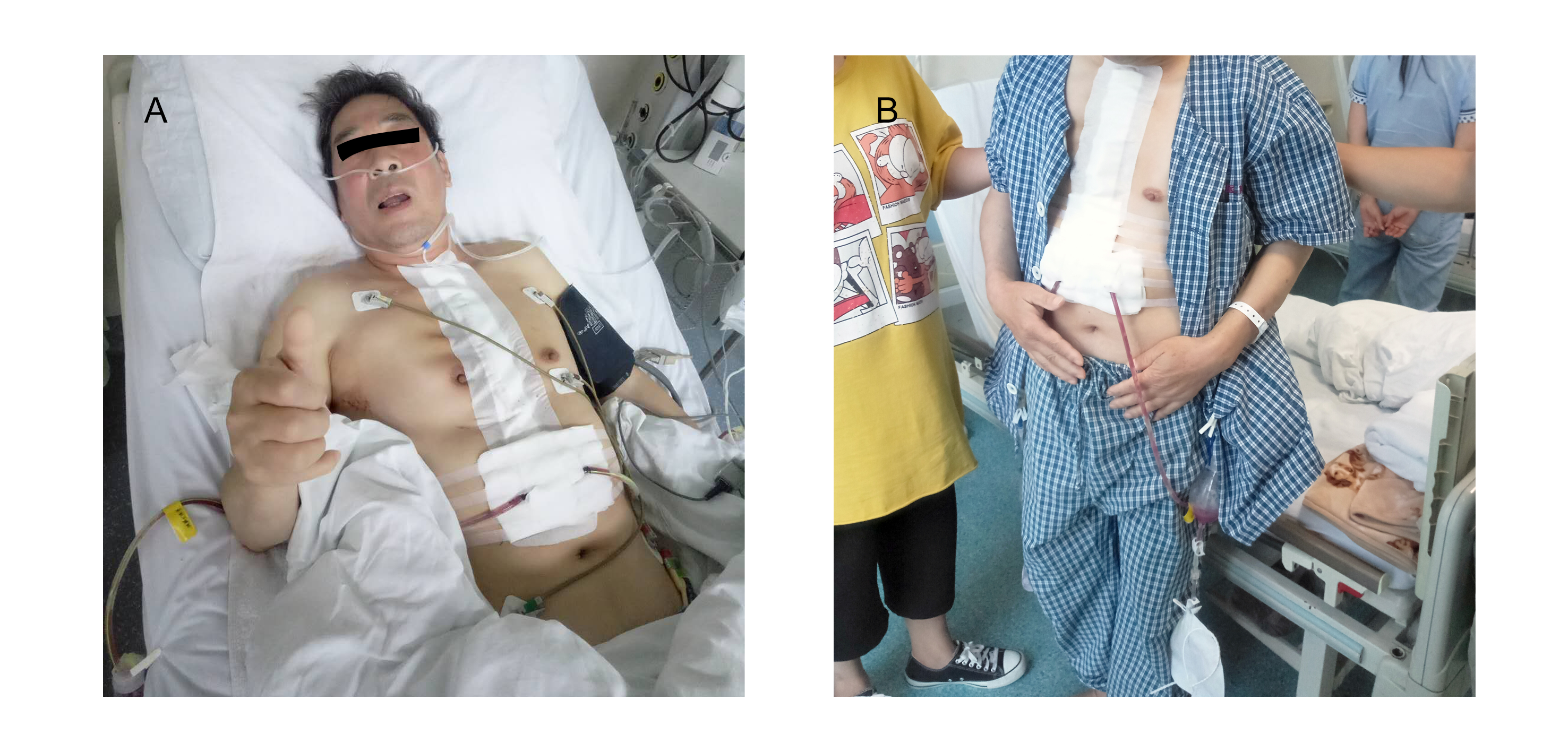

Supplement: Supplementary file 1 — Supplementary Figure 1 (a) The patient's condition on the first day after surgery; and (b) the condition of the patient on the seventh day after the operation. [file TCA-11-2709-s001.tif]
